# Deletion of ER-retention Motif on SARS-CoV-2 Spike Protein Reduces Cell Hybrid During Cell-cell Fusion

**DOI:** 10.21203/rs.3.rs-380389/v1

**Published:** 2021-04-09

**Authors:** Chih-Hsiung Chen, Saiaditya Badeti, Jong Hyun Cho, Alireza Naghizadeh, Xuening Wang, Dongfang Liu

**Affiliations:** Rutgers New Jersey Medical School; Rutgers New Jersey Medical School; Rutgers Biomedical and Health Sciences; Rutgers New Jersey Medical School; Rutgers New Jersey Medical School; Rutgers New Jersey Medical School

**Keywords:** COVID-19, Spike protein, syncytia, cell fusion, ACE2, cell hybrid

## Abstract

The novel SARS-CoV-2 has quickly become a global pandemic since the first reported case in December 2019, with the virus infecting millions of people to date. The spike (S) protein of the SARS-CoV-2 virus plays a key role in binding to angiotensin-converting enzyme 2 (ACE2), a host cell receptor for SARS-CoV-2. S proteins that are expressed on the cell membrane can initiate receptor-dependent syncytia formation that is associated with extensive tissue damage. Formation of syncytia have been previously observed in cells infected with various other viruses (e.g., HIV, Ebola, Influenza, and Herpesviruses). However, this phenomenon is not well documented and the mechanisms regulating the formation of these syncytia by SARS-CoV-2 are not fully understood. In this study, we investigated the possibility that cell fusion events mediated by the S protein of SARS-CoV-2 and ACE2 interaction can occur in different human cell lines that mimic different tissue origins. These cell lines were stably transduced with either wild-type (WT-S) S protein or a mutated variant where the ER-retention motif was removed (Δ19-S), or human ACE2 vectors. Different co-culture combinations of spike-expressing 293T, A549, K562, and SK-Hep1 cells with hACE2-expressing cells revealed cell hybrid fusion. However, only certain cells expressing S protein can form syncytial structures as this phenomenon cannot be observed in all co-culture combinations. Thus, SARS-CoV-2 mediated cell-cell fusion represents a cell type-dependent process which might rely on a different set of parameters. Recently, the Δ19-S variant is being widely used to increase SARS-CoV-2 pseudovirus production for in vitro assays. Comparison of cell fusion occurring via Δ19-S expressing cells shows defective nuclear fusion and syncytia formation compared to WT-S. This distinction between the Δ19-S variant and WT-S protein may have downstream implications for studies that utilize pseudovirus-based entry assays. Additionally, this study suggest that spike protein expressed by vaccines may affect different ACE2-expressing host cells after SARS-CoV-2 vaccine administration. The long-term effects of these vaccines should be monitored carefully.

## Introduction

Severe Acute Respiratory Syndrome-Coronavirus-2 (SARS-CoV-2) is a novel enveloped single-stranded RNA virus that causes Coronavirus-disease 2019 (COVID-19). It is part of the *Coronaviridae* family and since the initial report of the virus in 2019, COVID-19 has become a global pandemic. As of March 18th, 2021, there have been over 120 million confirmed cases of COVID-19 and over 2.6 million deaths, globally (WHO COVID-19 Dashboard. Geneva: World Health Organization, 2020. Available online: https://covid19.who.int/ (last cited: [03/18/21])). SARS-CoV-2 contains four types of structural proteins: nucleocapsid protein (N), membrane glycoprotein (M), envelope glycoprotein (E), and spike glycoprotein (S). Among these structural proteins, the S protein is highly conserved across human coronaviruses and is involved in viral attachment, fusion, and entry into cells [1]. S protein can mediate cell membrane fusion and viral entry into target cells upon binding to the host receptor, Angiotensin-converting enzyme 2 (ACE2), following proteolytic priming by TMPRSS2 [2, 3]. The structure of S protein consists of an N-terminal ectodomain, a transmembrane anchor, and a C-terminal cytoplasmic tail. The ectodomain contains the S1 subunit, which encodes the receptor-binding domain (RBD). RBD, as well as the S2 subunit which is necessary for membrane fusion, are key potential targets for treatment and vaccination strategies against COVID-19 [4–6]. Notably, the C-terminal cytoplasmic tail of the S protein encodes a presumptive endoplasmic reticulum (ER)-retention motif (known as KxHxx), which has previously been shown to enable the accumulation of SARS CoV-2 S proteins at the ER-Golgi intermediate compartment (ERGIC) and facilitate their incorporation into new virions [6, 7].

ACE2 is part of the renin-angiotensin-aldosterone system (RAAS) that controls blood pressure by regulating circulatory homeostasis and vascular functions [8]. It is a type I transmembrane protein that can act as both a peptidase and a viral receptor. ACE2 is mainly expressed on the cell surface of epithelial and endothelial cells of the heart, kidney, testes, lung, and gastrointestinal tract [4]. In RAAS, ACE2 acts to convert angiotensin-2, which can lead to vasoconstriction and inflammation, into active angiotensin homologs that has vasodilating and anti-inflammatory effects [9]. Therefore, ACE2 can regulate abnormal activation of the RAAS, preventing the development of hypertension, cardiac hypertrophy, and heart failure [8]. In COVID-19, ACE2 is the dominant host cell receptor for SARS-CoV-2 [10].

Of the four structural proteins of SARS-CoV-2, the S protein plays a key role in the process of ACE2 receptor recognition and cell membrane fusion [11]. Cell fusion events are either cell hybrids, in which chromosomes are combined into a single nucleus, or syncytia, where distinct nuclei are maintained within a single cytoplasm and plasma membrane [12]. Homotypic cell fusion occurs between cells of the same type. Heterotypic cell fusion occurs between cells of different types [13]. To demonstrate if S protein and ACE2 interaction can lead to cell-cell fusion in different scenarios, we generated cell lines expressing either wild-type S protein or Δ19-S conjugated to EGFP and hACE2 conjugated to mCherry. In Δ19-S, the 19 amino acids from the C terminus are deleted which results in the loss of S protein retention in the endoplasmic reticulum (ER). Different cell lines (293T, A549, K562 and SK-Hep1) expressing either S-WT-GFP or S-Δ19-GFP have been co-cultured with cells expressing ACE2-mCherry. These cell cultures were then observed using confocal microscopy to determine if cell fusion has occurred. The key findings in this study: 1) The interaction between S and ACE-2 can mediate cell fusion among different tissue-derived cell lines. 2) Interaction between the Δ19-S variant and ACE2 show defects in nuclear fusion during syncytia formation.

## Material And Methods

### Cell lines

293T cells, Sk-Hep1, K562 and NK92 cells were purchased from ATCC. A549 was a gift from Dr. Wei-Xing Zong (Rutgers-CINJ). K562 cells were cultured in RPMI-1640 (Corning) supplemented with 10% Fetal Calf Serum (FCS) and 1% Penicillin/Streptomycin (PS). 293T cells, A549, and SK-Hep1 cells were cultured in DMEM (Corning) supplemented with 10% FCS and 1% PS.

### Construction of plasmids

The SARS-Cov-2 Spike (or C terminal Δ19) gene was PCR amplified from the plasmids CHC3-pSFG_SARS-CoV-2 Spike or CHC4- pSFG_SARS-CoV-2 Spike Δ19 [14] with forward primer 5’- CTCACGCGTGCCACCATGGAGTTTGGGCTGAGCTGGC-3’ and reverse primer 5’- CTTTACTCATGGTGGACTTATCGTCGTCATCCTTGTAATCTC TAGAAGCG-3’ and were cloned into the pHR-EGFP vector (modified from Addgene plasmid #122147, which was linearized by PCR with forward primer 5’- GATGACGACGATAAGTC CACCATGAGTAAAGGAGAAGAACTTTTCACTG-3’ and reverse primer 5’-CAGCCC AAACTCCATGGTGGCACGCGTGAGAATTCTCG-3’) using the In-Fusion Cloning kit (Takara Bio) to generate CHC17-pHR_SARS-CoV-2 Swt_EGFP (or CHC-18 with Δ19,).

The hACE-2 gene was PCR amplified from the plasmid CHC21-pSFG_hACE-2 with forward primer 5’- GAATTCTCACGCGTGCCACCATGGAGTTTGGGCTGAGCTGGC-3’ and reverse primer 5’- CCTTTAGACACCATGGTGGACTTATCGTCGTCAT CCTTGTAAT CTCTA GAAAAG-3’ and were cloned into the pHR-mCherry vector (modified from Addgene plasmid #101221 which was linearized by PCR with forward primer 5’- CAAGGATGACGACGATAA GTCCACCATGGTGTCTAAAGGCGAGG-3’ and reverse primer 5’- CAGCTCAGCCCAAACTCCATGGTGGCACGCGTGAGAATTCTCG-3’ using the In-Fusion Cloning kit (Takara Bio) to make CHC16-pHR_hACE2_mCherry.

### Generation of stable cell lines with hACE-2-mCheny, SARS-CoV-2 Spike-full and -C terminal Δ19-EGFP.

293T cells were transfected with Invitrogen™ ViraPower™ Lentiviral Packaging Mix as followed: 2×10^6^ 293T cells were seeded the day before and mixed with 1ml Optimal MEM transfection solution with 45μl Genejuice (Millipore) containing 3.75μg pCMV-dR8.91, 2.5 μg pMD2.G-VSVG, and either 4.17 μg CHC16-pHR_hACE2_mCherry, CHC17-pHR_SARS-CoV-2 Swt_EGFP or CHC-18-pHR_SARS-CoV-2 S-Δ19_EGFP at RT for 15 mins, and then incubated with fresh D10 medium (10% FBS in DMEM without antibiotics) at 37°C and 5% (v/v) CO_2_ for 12 hours. Transfected 293T cell media was changed after 24 hours and incubated for another 48–72 hours. The lentivirus supernatant was harvested, filtered (0.45 μm filter Millipore) and transduced into 293T, A549, HepG2, and SK-Hep1 cells with serum-free DMEM for 12 hours. The transduced cell media was changed with fresh complete antibiotic-containing D10 medium for another 48–72 hours. Transduced cells were flow-sorted by GFP/mCherry expression, or protein expression determined by anti-Spike protein RBD domain antibody (rabbit IgG, Sino Biological) or anti-hACE2 antibody (goat IgG, R & D Systems) followed by fluorophore-conjugated goat anti-rabbit (Invitrogen) or donkey anti-goat secondary antibody (Jackson Immuno Research). Co-culture transduced cell lines were performed as listed in Table 1.

### Confocal microscopy imaging

Cells were co-cultured in glass chamber slides at a concentration of about 5×10^5^ cells/mL for 24 hours at 37°C. Cells were then fixed with 4% paraformaldehyde in phosphate-buffered saline (PBS) for 20 minutes at room temperature and stained with DAPI. The fluorescence images were obtained using a confocal microscope.

### Total intensity and MFI quantification of confocal images

The cell images are 4D, two-channel images, X (channel 1) and Y (channel 2). Each channel has Z number of slices to provide 3D image stacks of the cells. First, we identified the best slice based on total intensity of slide profiles. In order to obtain the quantifiable parameters of the cells in selected slices we used nuclei segmentation with multi-scale cell instance segmentation for channel X [15]. This method is an advanced form of artificial neural network that provides automated object detection and semantic segmentation for nuclei cells [16]. The network is already trained for similarly shaped cell objects to detect cells accurately. A sample of detected objects will show with rectangular shapes around detected cells, then show samples of segmented objects with different color masks on detected cells.

In order to find the contours over cells and perform final quantification, image processing is done with Python and OpenCV library. The OpenCV can obtain contours from the collection of segmented cells. The drawing contour function allows the thickness of the contours to be set manually, which determines the membranes of the cells. Image will show the drawn contours over the detected cells. Two types of quantifications: total signal intensity of the membranes and total signal intensity of cytosols were calculated. The contours represent membranes, and segmented cells with exclusion of contours, represent cytosols. The mean fluorescence intensity (MFI) was calculated by dividing the total signal intensity over the area within regions of interest (ROIs). The ROIs for membranes were calculated by the pixel area of contours for each individual cell. The ROIs for cytosols were calculated by the pixel area of segmented cells with the exclusion of contours for each individual cell.

## Statistical analysis

All data calculations and statistics were performed using MS Excel. Figures and graphs were created using GraphPad Prism 8. Statistical significance between different groups was calculated using Student’s T-test.

## Results

### Establishment of S-WT-EGFR S-Δ19-mGFP effector cell lines and hACE2-mCherry target cell lines.

**Figure 1:**
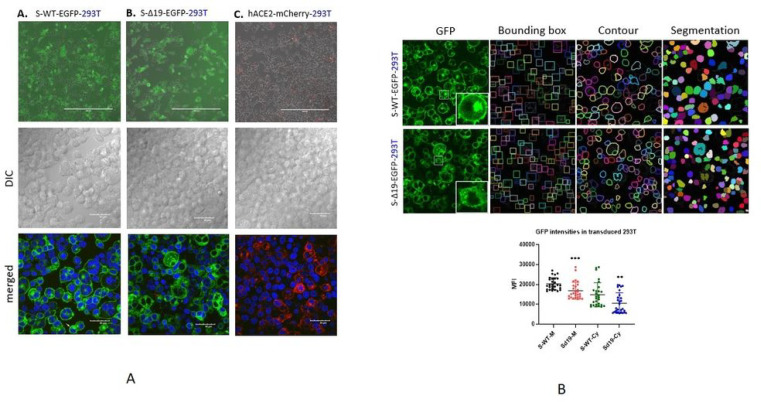
Representative images of 293T cells transduced with S-WT-GFR S- Δ19-GFP and hACE2-mCherry vectors. (A) Top panels show the expression of enhanced GFP and mCherry in 293T cells under reverse fluorescence microscope following transduction by lentiviral vectors. Bottom two rows show successful expression of S-WT-GFP S Δ19-GFP or hACE2-mCherry in 293T cells by high resolution confocal microscopy. Middle row represents DIC images. Bottom row represents merged confocal image (DAPI, blue; S-WT-GFP or S-Δ19-GFP green; hACE2-mCherry, red). Scale bar equals 40 μm. (B) The mean intensities of enhanced GFP on cell membrane or cytosol were measured using multi-scale cell instance segmentation framework method, then plotted in a bar chart. P=0.0001 when compare intensities of S-WT-M group with intensities of S- Δ19-M; P=0.0060 when compare intensities of S-WT-Cy group with intensities of S- Δ19-Cy using nonparametric T-test. (M=membrane, Cy=Cytosol).

Various cell lines were transduced with the indicated plasmids. Then, cells were sorted based on high expression and verified by either enhanced GFP or mCherry fluorescence using a widefield fluorescence microscope and filter combinations optimized for the appropriate fluorescent proteins (Fig. 1A). To confirm the intensities of GFP of either wild-type S protein or Δ19 S protein in 293T cells, the images were processed using the multi-scale cell instance segmentation framework method. Then the intensities of membrane-bound S protein or cytosol-bound S protein were plotted with mean intensities of all cells. The WT-S protein were mainly located on the cell membrane and ER region surrounding the nucleus. The S-Δ19 bar chart demonstrated less intensities compared to wild-type S protein (Fig. 1B). Thus, we successfully established different types of cell lines with S-WT-EGFP S-Δ19-mGFP and hACE2-mCherry expression.

### Co-culture of S-WT-EGFP 293T cells and hACE2-mChenry 293T cells forms hybrid cell fusion.

**Figure 2:**
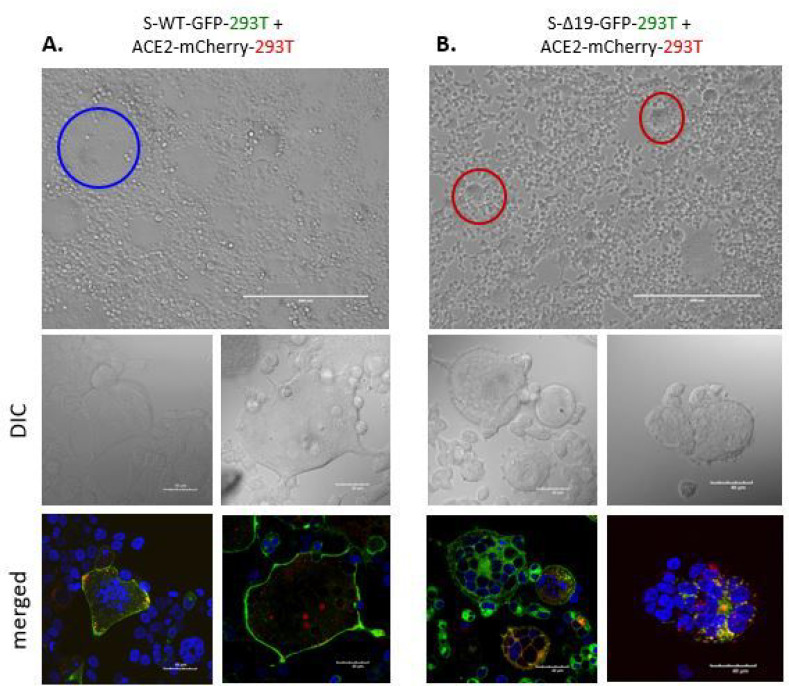
Spike protein mediates cell fusion in transduced 293T cells. (A) Representative images of cell-cell fusion in co-cultures of either S-WT-293T and hACE2–293T cells with EVOS FL color image systems (top panel) or confocal microscope (bottom panel) (DAPI, blue; S-WT-GFP or S-Δ19-GFP green; hACE2-mCherry, red). (B) Representative images of cell-cell fusion in different co-cultures of S-Δ19–293T, and hACE2–293T cells with EVOS FL color image systems (top panel) or confocal microscope (bottom panel) (DAPI, blue; S-WT-GFP or S-Δ19-GFP green; hACE2-mCherry, red). Scale bar equals 40 μm.

To establish a method for visualizing Spike protein-mediated cell-cell fusion, we first designated 293T cells expressing wild-type S or S-Δ19 conjugated to enhanced green fluorescent protein (EGFP) as effector cells and 293T cells expressing the human ACE2 conjugated to mCherry as target cells. Both cells were co-cultured together in glass chamber slides. In the co-culture combinations performed, we could observe cell fusion events and larger-than-normal cells (the controls). Using confocal microscopy, we could visualize fused cells in detail with more than ten S-EGFP and hACE2-mCherry cells per sample fused with intact cell membranes and containing multiple lysed nuclei (Fig. 2). However, in 293T-S-Δ19-GFP with 293T-ACE2-mCherry co-cultures, we observed fused cells that had individual, non-fused nucleus. This phenomenon may be explained by the deletion of the ER retention motif in S-Δ19 cells. Therefore, in S-Δ19 cells, translated S proteins are not retained in the ER, but instead trafficked to the cell surface or secreted as viral particles. Because there is no Spike protein retained in the ER, we hypothesize that there will be minimal Spike protein and hACE2 interaction juxtaposed to the ER of contacting cells and therefore no fusion of captured nuclei. However, Spike WT expressing cells have normal ER signaling retention of S proteins. Therefore, when ACE2 and S proteins are both present in a fused cell, it may lead to ER fusion which can lead to clumped or fused nuclei due to the close proximity between ER and nucleus.

### Cell fusion in Spike or hACE2 expressed A549 cells.

**Figure 3:**
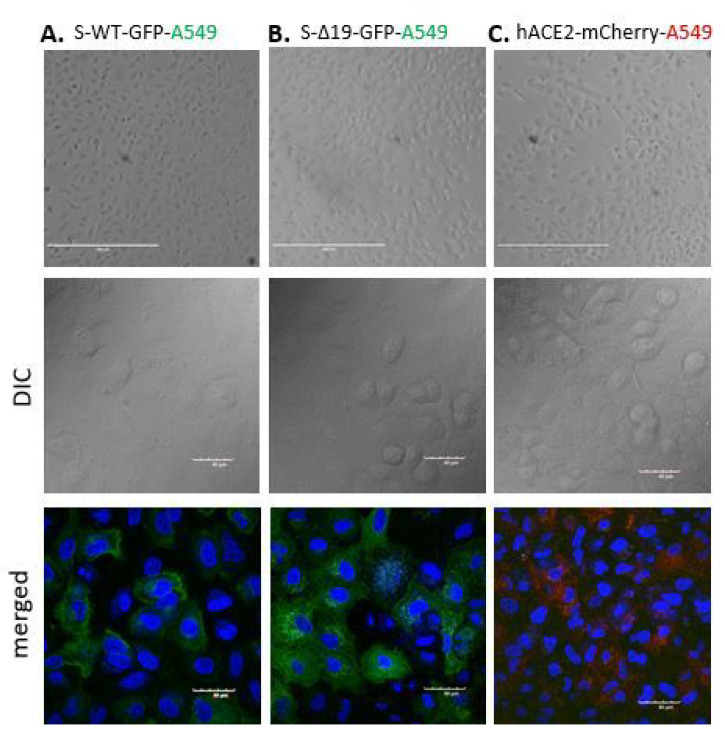
Representative images of A549 cells transduced with S-WT-GFR S-Δ19-GFP and hACE2-mCherry vectors. Top panels show transduced A549 cells under EVOS FL color image systems, Scale bar equals 400 μm. Bottom two rows show successful expression of S-WT-GFP S Δ19-GFP or hACE2-mCherry in A549 cells by high resolution confocal microscopy. Middle row represents DIC images. Bottom row represents merged confocal image (DAPI, blue; S-WT-GFP or S-Δ19-GFP green; hACE2-mCherry, red). Scale bar equals 40 μm.

**Figure 4:**
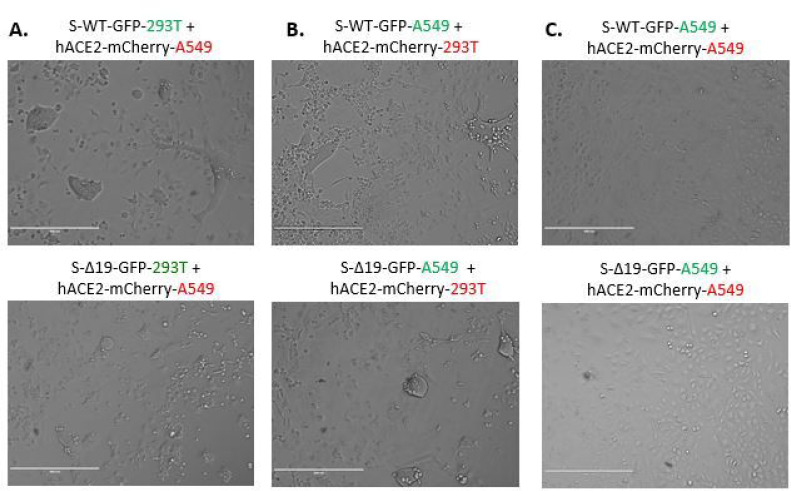
Co-culture of transduced A549 cells and transduced 293 cells induced syncytia formation. (A) Representative images of cell-cell fusion in co-culture of either S-WT-293T or S-Δ19–293T with hACE2-A549 cells using EVOS FL color image systems. (B) Representative images of cell-cell fusion events in co-culture of either S-WT-A549T or S-Δ19-A549T with hACE2–293T cells. (C) Images of cell-cell fusion in co-culture of either S-WT-A549T or S-Δ19-A549T with hACE2-A549 cells. Scale bar equals 400 μm.

**Figure 5:**
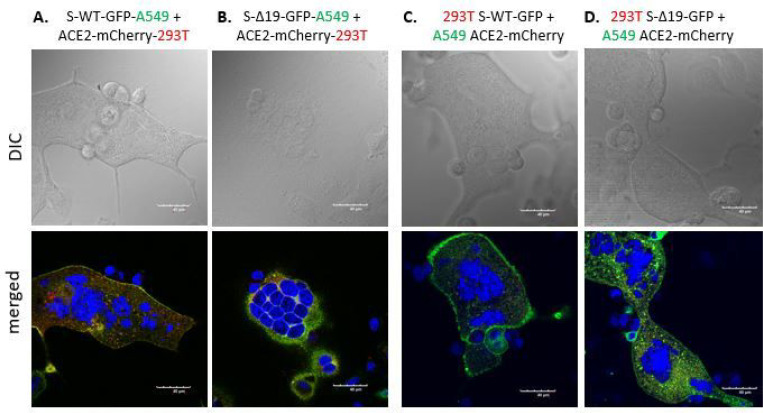
Spike protein mediates cell fusion in transduced A549 and 293T cells. (A & B) Immunofluorescent images of syncytial formation when co-culture S-WT-A549 or S-Δ19-A549 with hACE2–293T cells were obtained by confocal microscope. (C & D) Similarly, immunofluorescent images of hybrid or syncytial formation when co-culture S-WT-293T or S-Δ19–293T with hACE2-A549 cells. Scale bar equals 40 μm.

Next, we performed the same experiment with A549 cells, a human alveolar basal epithelial adenocarcinoma cell line [17], expressing either S proteins or ACE2, and confirmed the expression of either protein by confocal imaging (Fig. 3). The co-culture of hACE2-A549 with S-WT-293T demonstrated hybrid cell fusion formation, while mixed culture of S-Δ19–293T with hACE2-A549 only manifested few smaller cell fusion events after 24 hours (Fig. 4B). Co-culture of S-WT-A549 or S-Δ19-A549 cells with hACE2–293T cells showed similar results (Fig. 4C). These results were confirmed with high resolution confocal microscopy (Fig. 5). However, A549-S-WT or S-Δ19-GFP cells co-cultured with A549-ACE2-mCherry exhibited few cell fusion formations (Fig. 4C). The number of fused cells we could observe were much less compared to other cell co-culture combinations which may indicate that other signaling checkpoints are necessary for Spike protein-mediated cell fusion to occur.

### Cell fusion in Spike or hACE2 expressed K562 cells.

To investigate SARS-CoV-2’s impact on the hematopoietic system, we transduced the S protein and hACE2 onto K562 cells. K-562 is a human erythroleukemia line derived from a 53-year-old female chronic myelogenous leukemia patient in blast crisis [18]. K562 cells can develop characteristics similar to early-stage erythrocytes [19].

**Figure 6:**
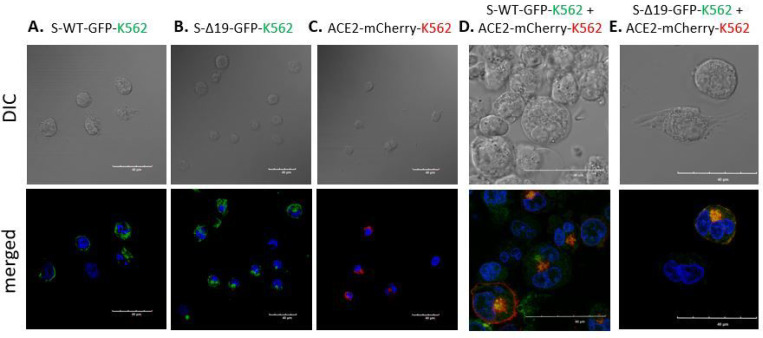
Representative images of K562 cells transduced with S-WT-GFR S-Δ19-GFP and hACE2-mCherry vectors. Expression of S-WT-GFP (A), S Δ19-GFP (B), or hACE2-mCherry (C) in K562 cells by high resolution confocal microscopy. Top row represents DIC images. Bottom row represents merged confocal image (DAPI, blue; S-WT-GFP or S-Δ19-GFP green; hACE2-mCherry, red). (D & E) Images of cell-cell fusion in co-culture of either S-WT-K562 or S-Δ19-K562 with hACE2-K562 cells. Scale bar equals 40 μm.

**Figure 7:**
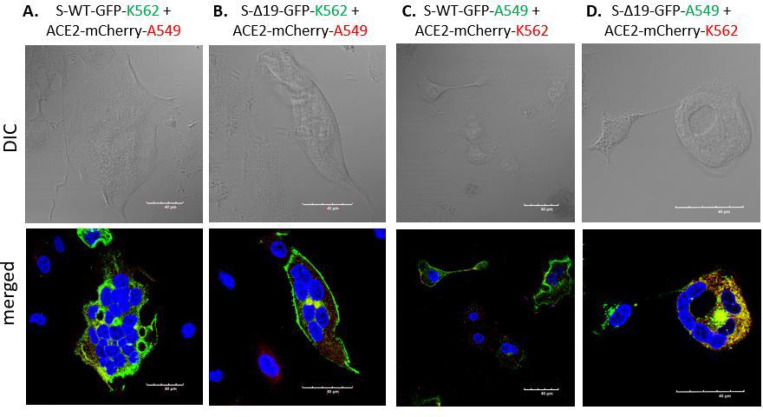
Spike protein mediates cell fusion in transduced K562 and A549 cells. (A & B) Immunofluorescent images of hybrid or syncytial formation when co-culture S-WT-K562 or S-Δ19-K562 with hACE2-A549 cells were obtained by confocal microscope. (C & D) Immunofluorescent images of cell fusion when co-culture S-WT-A549 or S-Δ19-A549 with hACE2-K562 cells. Scale bar equals 40 μm.

Co-culture of S-WT or S-Δ19 K562 with hACE2 K562 cells showed cell fusion in a few cells (Fig. 6). However, fluorescence images of other co-cultures using A549 and K562 cells showed that while there were indeed cell fusions, albeit with different patterns, fused hACE2 cells expressed a combination of both fused and unfused nuclei when co-cultured with S-WT and S-Δ19 cells (Fig. 7). We speculate that nuclear fusion in fused cells happens in a time-dependent manner where individual nuclei present in fused cells at the beginning of the process eventually fuse together into one large nucleus as the intact individual nuclei begin to disintegrate. Interestingly, we also observed that S-Δ19 cells seem to express the S protein more strongly in the cytosol than on the cell membrane. Further studies would have to look more into the expression level and trafficking of the S-Δ19 protein, as it compares to the original S-WT protein.

### Interaction of Spike and hACE2 mediated-cell fusion events between different Cell types.

**Figure 8:**
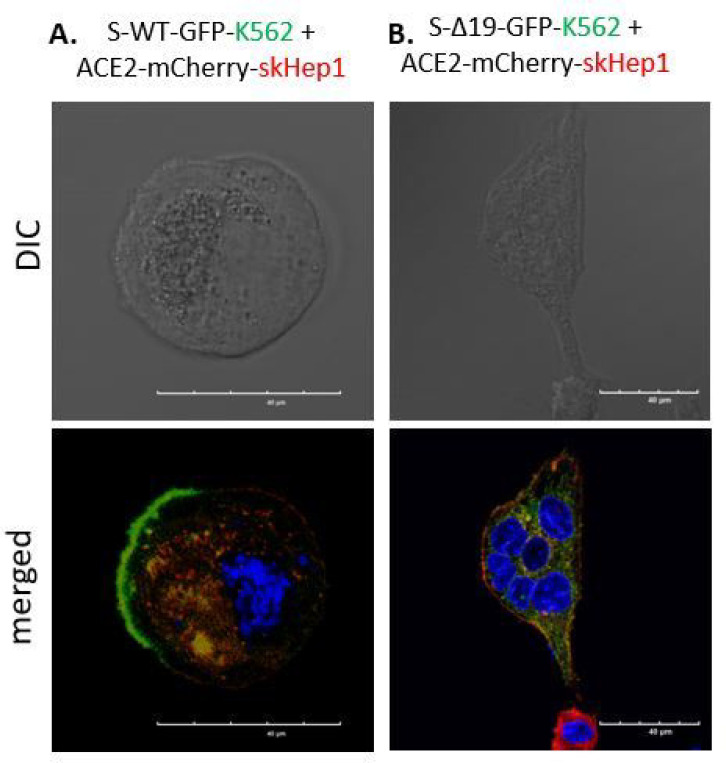
Spike protein mediates cell fusion in transduced K562 and SK-Hep1 or NK92MI cells. (A & B) Immunofluorescent images of hybrid or syncytial formation when co-culture S-WT-K562 or S-Δ19-K562 with hACE2-SK-Hep1 cells were obtained by confocal microscope. Scale bar equals 40 μm.

Studies showed that chronic liver disease might predispose to poorer outcomes following SARS-CoV-2 infection due to an altered immune profile and systemic inflammation [20]. We investigated whether similar cell fusion events could take place in liver cancer cells by co-culturing S-WT/S-Δ19 K562 cells with hACE2-SK-Hep1 cells. S-WT-K562 induced syncytia formation when combined with hACE2-SK-Hep1 cells, and co-culture of S-Δ19-K562 with hACE2-SK-Hep1 demonstrated different fusion patterns to the wild-type S protein (Fig. 8A&B).

Furthermore, to demonstrate that the observed giant cells are indeed fused cells and not just clumps of individual cells, we performed flow cytometry on 293T cells following 24 hour co-cultures (S-WT-GFP/S-Δ19-GFP with hACE2-mCherry) and observed that there were some cell populations that were double positive for S-WT-GFP/S-Δ19-GFP and hACE2-mCherry (data not shown). Cell fusion upon S protein and ACE2 contact may be of interest to the scientific community as there could be a potential possibility that efficacious COVID-19 vaccines induce transient or permanent cell fusion events within vaccinated individuals. Lysis of fused cells may damage the affected tissues in the long run.

## Discussions

Cell-cell fusion can be triggered between virus-infected cells and neighboring target cells to form enlarged either cell hybrids or syncytia. There are several groups of viruses, such as HIV, influenza, herpesviruses and SARS-CoV-2 that can induce such cell-cell fusion [11, 21–23]. In vivo study of the roles of virus-induced cell fusion is important in understanding the virus transmission and its contribution to viral pathogenesis.

Spike protein-induced cell fusion allows SARS-Cov-2 virus infected cells to merge with other cells through cell-cell fusion without the need to bud and produce free virus. Fusion can not only damage neighboring cells in proximity with infected cells that express membrane spike protein, but it can also influence distant organ tissue through cell-cell fusion of infected cells with cells from different organs. Our results demonstrate that hybrid cell fusion formation represents a cell type-dependent process. Furthermore, deletion of the last 19 amino acids of spike protein, which contain the ER-retention motif, reduce hybrid cell formation. The summary of co-culture combinations on hybrid or syncytia formation are listed in Table 2.

The timeline of cell fusion events is different depending on the expression level of S proteins. Nuclear fusion [13] may be a gradual process beginning with accumulation of individual nuclei in fused cells, culminating in the creation of one large nucleus, and resulting in complete nuclear disintegration and mixing of genetic material. We also observed that truncated S-Δ19 expressed cells seem to have more S protein distributed in the cytosol than on the cell membrane, while wild type S protein is mainly expressed on transduced cell membranes and in the ER region surrounding the nucleus. S-Δ19 expressed cells still have a functional binding domain in the S1 region, but the ER-retention motif in the C-terminal region, has been removed. This deletion not only reduced S protein retention in the ER region, it also reduced the occurrence of nuclear fusion. Further studies need to investigate trafficking of truncated S protein to better understand machinery involved in cell fusion.

This distinction between the modified and wild-type S protein may also have downstream implications for studies that utilize pseudovirus-based entry assays. Additionally, the data in this study suggest that spike protein may affect hACE2-expressing host cells differently after COVID-19 vaccine administration. The long-term effects of these vaccines should be monitored, carefully.

## Figures and Tables

**Table 1:** Co-culture combinations of transduced cell lines

Effector cells	Target cells
S WT-GFP / S D19-GFP – 293T	hACE2-mCherry – 293T
S WT-GFP / S D19-GFP – 293T	hACE2-mCherry - A549
S WT-GFP / S D19-GFP -A549	hACE2-mCherry – 293T
S WT-GFP / S D19-GFP -A549	hACE2-mCherry - A549
S WT-GFP / S D19-GFP -A549	hACE2-mCherry - K562
S WT-GFP / S D19-GFP -K562	hACE2-mCherry - A549
S WT-GFP / S D19-GFP -K562	hACE2-mCherry - K562
S WT-GFP / S D19-GFP -K562	hACE2-mCherry - SK-Hep1

**Table 2:** Hybrid or syncytia formation in co-culture combinations of transduced cell lines

Effector cells	Target cells	hybrid	Effector cells	Taget cells	Syncytia
S WT-GFP-293T	hACE2-mCherry – 293T	**++++**	S D19-GFP-293T	hACE2-mCherry – 293T	**+++**
S WT-GFP-293T	hACE2-mCherry - A549	**++**	S D19-GFP-293T	hACE2-mCherry - A549	**++**
S WT-GFP-A549	hACE2-mCherry – 293T	**++++**	S D19-GFP-A549	hACE2-mCherry – 293T	**+++**
S WT-GFP-A549	hACE2-mCherry - A549	**+/−**	S D19-GFP-A549	hACE2-mCherry - A549	**+/−**
S WT-GFP-A549	hACE2-mCherry - K562	**+**	S D19-GFP-A549	hACE2-mCherry - K562	**++**
S WT-GFP-K562	hACE2-mCherry - A549	**++**	S D19-GFP - K562	hACE2-mCherry - A549	**++**
S WT-GFP-K562	hACE2-mCherry - K562	**+**	S D19-GFP-K562	hACE2-mCherry - K562	**+**
S WT-GFP-K562	hACE2-mCherry-SKHep1	**++**	S D19-GFP-K562	hACE2-mCherry-SKHep1	**++**

The size of fused cells less than 20 uM defined as “+/−”; 20–40 uM as “+”, 40–80 uM as “++”, 80–120 uM as “+++”, larger than 120 uM as “++++”
